# Molecular-genetic characterization of human parvovirus B19 prevalent in Kerala State, India

**DOI:** 10.1186/s12985-021-01569-1

**Published:** 2021-05-05

**Authors:** Dayakar Seetha, Heera R. Pillai, Sai Ravi Chandra Nori, Sanu Ghosh Kalpathodi, Vineetha P. Thulasi, Radhakrishnan R. Nair

**Affiliations:** 1 Laboratory Medicine and Molecular Diagnostics Rajiv Gandhi Centre for Biotechnology (RGCB), Thiruvananthapuram, 695585 India; 2 Teagasc Food Research Centre, Moorepark, Fermoy, Co. Cork Ireland; 3 School of Microbiology, University College Cork, Cork, Ireland

**Keywords:** Human Parvovirus B19V, VP1/2, Nested PCR, Real-time PCR and virus evolution

## Abstract

**Background:**

Human parvovirus B19V is a DNA virus, and a member of the family *Parvoviridae,* that causes various clinical manifestations, from asymptomatic to persistent infection that is associated with different autoimmune diseases. The parvovirus B19 evolves with a very high mutation rate that is closer to those of existing RNA viruses. Globally circulating B19V is currently classified into three genotypes, but their distribution is not spatially and temporally correlated. Except for a few recent reports on B19V entry into the human host and its genetic diversity, there is a lack of sufficient studies on this virus from distinct geographical locations and no clear understanding of its evolution has been documented.

**Methods:**

To better understand the evolution of the Human parvo B19V virus from India's southern part, a geographically distinct location with no reports of B19V genomes, we have screened for B19V in 456 suspected cases using VP1/2 surface marker genes, and its characteristics were studied in detail. Amongst 456 clinically suspected B19V samples, 7.2% (33/456) were found positive by nested PCR (nPCR) were subsequently validated by real-time PCR, Sanger sequencing, and metagenome analysis.

**Results:**

Human parvovirus B19 infection was shown among 33 of 456 patients when tested by nPCR; 30 among these were also positive by qPCR and were subsequently confirmed by sequencing 75% nPCR positive samples and 76% qPCR positive samples were from patients with age. ≥ 50 years respectively (Additional file 1: Table S1). The complete VP1/2 gene assembly from the South Indian strain showed three novel mutations (T122A, V128I, I283V), which might significantly impact the stability and virulence of the B19V virus circulating in this part of the world. These mutations might be crucial for its adaptive evolutionary strategies facilitating the spread and infectivity potential of the virus. In maximum likelihood phylogeny of VP1/2 sequences, the South Indian B19V strain forms a separate clade closer to the existing genotype two strains circulating worldwide.

**Conclusion:**

Our study contributes to a better understanding of the human parvovirus's genetic and evolutionary characteristics in South India. Also, it highlights the possibility that a positive selection pressure acting on VP1/2 could increase the survival and replication capabilities of the viruses.

**Supplementary Information:**

The online version contains supplementary material available at 10.1186/s12985-021-01569-1.

## Introduction

Human parvovirus B19V (B19V) is a DNA virus of the Parvoviridae family and genus Erythroparvovirus [1]. B19V, an omnipresent pathogen, causing a broad spectrum of clinical manifestations such as childhood rash erythema infectiosum, arthralgias, and in pregnant women, it can cause fetal death (hydrops fetalis) [2]. The infections of B19V are mild or asymptomatic and can lead to transient or persistent erythroid aplasia and aplastic crisis in people with underlying hematological disorders [3]. Management of this viral infection is limited to symptomatic treatment sadly due to the lack of specific antiviral drugs/vaccines [4, 5].

B19V virus has a single-stranded DNA genome of 5.6 kb, which codes for three proteins: a nonstructural protein (NS1) and two viral capsid proteins VP1 and VP2. VP2, a major capsid protein, has a pivotal role in the viral assembly of the B19 virus and is identified as an attractive molecular target for structure-based drug discovery [6]. NS1 protein plays a significant role in viral replication, which is a pleiotropic nuclear phosphoprotein. It is a multi-functional protein that aids in cellular transcription, virus replication, cell death induction, and cellular promoters' transactivation [7]. VP1, a minor capsid protein, has the same amino acid sequence as VP2, plus an additional 227 amino acids at the N-terminus called the VP1-unique region (VP1u). The VP1u exhibits relatively high sequence variability in persistently infected individuals and plays an essential role in eliciting specific immune responses [8]. Substitution rates of B19V are unusually high, in the range of 1.0–4.0 × 10^−4^ nucleotide substitutions per site per year, which is more similar to substitution rates of RNA viruses than either single-stranded or double-stranded DNA viruses [9], which makes this virus unique.

The human B19V is classified into three distinct genotypes based on the NS1-VP1u region. Genotype 1 has a ∼10% sequence divergence from genotypes 2 and 3, and genotypes 2 and 3 have a ∼5% sequence divergence between each other [10]. Genotypes 1 and 3 are further categorized into subtypes a and b with the divergence of about 5% [11]. The distribution of the three genotypes is not spatially and temporally uniform: genotype 1 has a worldwide distribution [12], Genotype 2 is found mainly in elderly adults in northern Europe [13], and Genotype 3 is found in Sub-Saharan and West Africa, South America, and France [14]. Many reports are available from distinct geographical areas, including Europe, Asia, South America, and Africa; reports on B19V genotypes from India is not yet available. Despite the recent reports of viral entry and genetic diversity, there is still a lack of sufficient data from distinct geographical locations to understand B19V evolution. Here, we performed assembly of B19V genome using metagenomics sequencing and studied sequence variation and the phylogenetic relationship of B19V isolated from South India to other global strains. Our study reports a distinct genotype for human B19V isolated from South India, with a 98.78% sequence similarity to currently circulating Genotype 2 strains. The study further helps to understand the nature of parvovirus evolution.

## Materials and methods

### Clinical specimen collection

The Laboratory Medicine and Molecular Diagnostic facility of Rajiv Gandhi Center for Biotechnology in Kerala, South India, samples containing tentatively higher viral genome equivalents as detected by nPCR and qPCR( high viral load) were collected. Further, the samples were sequenced, genetic and structural characteristics of the samples were analyzed.

### Extraction of DNA, nPCR, and sequencing

DNA was extracted from 200 µl of EDTA blood by a spin-column procedure (QIAamp DNA Mini Kit™, QIAGEN, and Germany). All samples were also tested for β-actin by PCR assay. 5 µl of DNA was used as a template in nested PCRs for the B19V NS1 gene. The primers for VP1/2, NS1 regions were designed by the Primer 3 program (Whitehead Institute for Biological Research) and analyzed for erythrovirus specificity using the NCBI blast. For first (40 cycles) and second (40 cycles) rounds consisted of initial denaturation of 95 °C for 6 min, 95 °C for 30 s, 55 °C for 30 s, and 72 °C for 30 s. Amplifications were carried out using the using Gene Amp® PCR system 9700 (Applied Biosystems, USA). Electrophoresis amplified products were analysed using 1.5% agarose gel containing ethidium bromide and visualized under UV illumination (E-gel Imager, Life technologies, USA) [15].

**Fig. 1:**
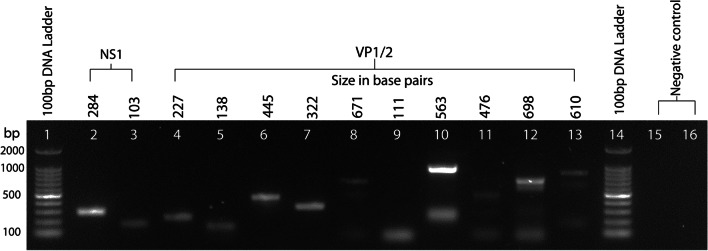
Analysis of DNA fragments obtained by nested PCR on the unique conserved regions of VP1/2 gene of human B19V. Parvovirus B19V PCR and amplified products were electrophoresed in a 1% agarose-TAE gel and stained with ethidium bromide solution for visualizing using the E-gel imager (Life technologies, USA) gel documentation system. Lane 2 and 3: 284 and 103 bp amplified products of NS1 gene; Lane 4–13: 227, 138, 445, 322, 671, 111, 563, 476, 698 and 610 bp amplified products of VP1/2 gene; Lane 1 and 14 100 bp DNA ladder; lane 15 negative patient sample and 16 negative control. Hepatitis B virus positive clinical samples were used as a negative control for testing primer specificity

Representative PCR products from the nPCR were confirmed using Sanger sequencing using the same forward primers used in the PCR. The reverse primer was used in cases where the forward primer reaction failed for the sequencing protocol. Agarose gel-purified fresh PCR products were subjected to cycle sequencing using the Big Dye Terminator v3.1 Cycle Sequencing Kit (Applied Biosystems, USA) according to the manufacturer’s instructions. Sequences were read using the ABI PRISM 7900 sequencer (Applied Biosystems, USA) and results were analyzed using sequencing analysis 5.2 software (Applied Biosystems, USA). The obtained sequences were subjected to the NCBI BLAST analysis (http://blast.ncbi.nlm.nih.gov/) for sequence homology analysis (Fig. 1).

### Real-time PCR (qPCR)

Real-time PCR was performed using Real Star Parvovirus B19V system, which includes a heterologous amplification system (internal control) used as a PCR inhibition control to detect human B19V from the clinical samples. Ten microliters template was used for PCR amplification. Thermal cycling conditions for qPCR were 95 °C for 10 min for initial denaturation,45 cycles of 15 s at 95 °C, 1 min at 58 °C (FAM, JOE),72 °C at 10 s as per the manufacturer recommendations using the Rotor gene 5plex HRM real-time platform (QIAGEN, Germany) [15].

### Library preparation and metagenome sequencing

Whole-genome libraries were prepared using the NEB Ultra II kit (Cat. No: E7645L)**.** In brief, the total DNA extracted from EDTA blood was subjected to a sequence of enzymatic steps to fragment them, the ends of these fragments were repaired enzymatically, and tailing with dA-tail was done followed by ligation of adapter sequences. The adapters ligated fragments were then cleaned up using Agencourt AMPure XP bead (Cat.No: A63882)**.** These fragments were further indexed using limited cycle PCR to generate final libraries for paired-end sequencing. The raw reads were subjected to quality checks that include Base quality score distribution, Sequence quality score distribution, Average base content per reading, and GC distribution in the reads. The low-quality sequence reads are excluded from the analysis. The adapter trimming was performed using trimmomatic[16]. Prepared libraries were quantified using Qubit High Sensitivity reagent. The obtained libraries were diluted to a final concentration of 2 nm in 10 µl and were subjected to cluster amplification. Once the cluster generation was completed, the flow cells were loaded onto the sequencer. The sequencing was carried out in Hi Seq X10 to generate 2X150 bp sequence reads at 100X sequencing depth (~ 1 GB). A minimum of 75% of the sequenced bases was of Q30 value. Sequenced data were further pre-processed to generate FASTQ reads (https://www.bioinformatics.babraham.ac.uk/projects/fastqc/) [17].

### Denovo assembly of Parvovirus B19V from metagenome

The Illumina processed reads were aligned to reference Human Genome (GRCh37/hg19) using bowtie tool [18] and 99% of reads got aligned with the human reference genome. The remaining 1% unaligned reads were mapped with the human B19V reference genome (KX752821), and reads that map with B19V reference were taken for denovo assembly using Velvet assembly tool [19] with the default parameters. From the resulting three contigs, protein-coding genes were retrieved using Prodigal [20]. Predicted genes were BLAST searched against the human B19V reference genome (KX752821) using BLASTP (https://blast.ncbi.nlm.nih.gov), best hits with e-value ≤ 1e−05, query-coverage > 70%, 95% similarity were considered for further downstream analysis.

### Sequence and phylogenetic analyses

Seventy-five amino-acid full-length sequences of human B19V VP1/2 previously reported from different geographies were retrieved from NCBI and multiple sequence alignment was done using MAFFT [21] with options maxiterate (1000), global pair/ginsi. Identified amino acid mutations were mapped into VP1/2 of human B19V reference structure (PDB ID: 1s58) using pymol [22] visualization program. A Maximum Likelihood phylogenetic tree of the VP1/2 sequence and its homologs was constructed by IQ-Tree [23] using "JTT + G4" as substitution model with bootstrap (n = 1000) for phylogeny. Immunogenicity analyses of the VP1/2 sequence were done by the tool Epitopia [24]. Epitopia ranks individual amino acids according to their potential of eliciting a humoral immune response. Selection pressure analysis was done using the HyPhy software package (http://www.hyphy.org/). Overall selection pressure, measured as the mean ratio of non-synonymous (dN) to synonymous (dS) substitution were estimated using different likelihood approaches seen inside Hyphy suite: the Single Likelihood Ancestor Counting (SLAC), Fixed-Effects Likelihood (FEL) and Random-Effects Likelihood (REL) methods [25]. We used the MEME program, which identifies instances of episodic and positive selection at individual sites [26].

### Homology modelling

The PDB structural file of the VP1/2 structure of reference human B19V (PDB: 1s58) was used as a template for homology modeling. The mutant VP1/2 gene was modeled by I-TASSER tool [27] and the predicted structure was visualized using Pymol (Schrodinger, LLC). Various tools were used to predict the protein stability variation due to single-residue change: I-Mutant [28], ELASPIC [29] and mCSM [30]. The Relative Solvent Accessibility (RSA) of the predicted protein structure was predicted by Raptor-X's tool [31].

## Results

### Detection, quantification and sequencing of B19V

Amongst 456 clinically suspected B19V samples screened at the Laboratory Medicine and Molecular Diagnostics facility, of Rajiv Gandhi Centre for Biotechnology by nested PCR (nPCR), 7.2% (33/456) were found positive (Additional file 1: Table S1). The positive cases were further tested and validated by real-time PCR and Sanger sequencing. Three samples had less than the detectable limit of viral load for real-time PCR confirmation (Additional file 2: Figure S1). The BLAST data confirmed the amplified products to be that of the human paro virus B 19 (Fig. 1).

### Genetic and structural insights of VP1/2 full length sequences

Metagenomic sequencing and assembly of full-length VP1/2 sequence of B19V were performed from a sample that showed high viral load (66,754,059 IU/ml). Comparative analysis of VP1/2 full-length sequence of South-Indian B19V strain showed an average 98.78% sequence similarity to Genotype 2 strains. It offers an average sequence similarity of 98.13% with Genotype 3 and 97.97% with Genotype 1, respectively (Additional file 3: Table S2).

**Fig. 2:**
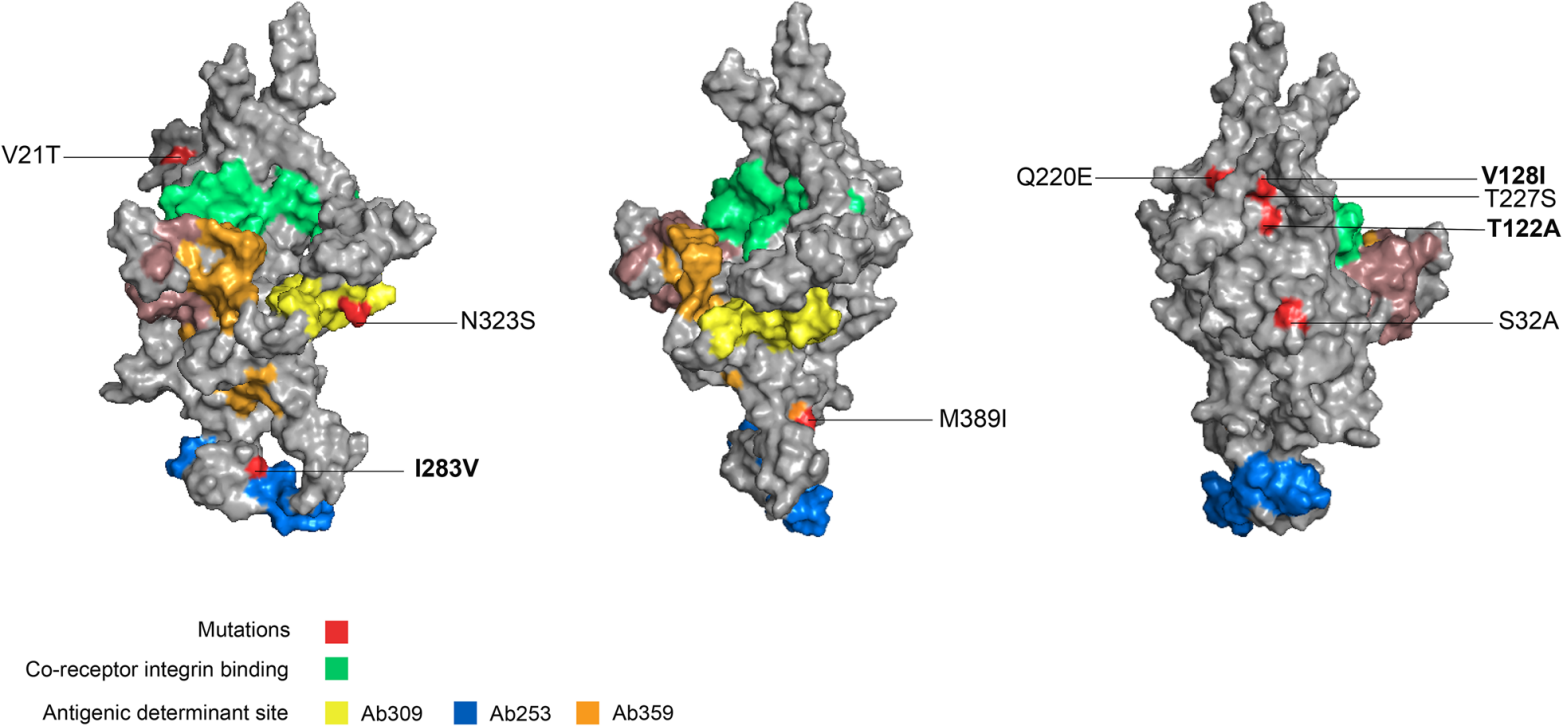
Atomic structure of VP1/2 capsid of parvovirus B19V showing mutated sites. The mutations unique to the South-Indian B19V strain are highlighted in bold

The mutated residues present in VP1/2 of South Indian strain are shown in red color (V21T, S32T, T122A, V128I, T227S, I283V, N323S, M389I, Q220E), out of which bold ones (T122A, V128I, I283V) are unique to South-Indian B19V strain. The modeled protein structure depicts a surface model, shown in three views, (a) first normal view, and the other two surface representations of a given structure in two different angles (b) 45° and (c) 120°. The co-receptor integrin-binding residues are (82–87, 168–188) colored in green. The epitope/antigenic determinant like Ab-253 residues are (253–272) represented in the blue square, Ab-309 residues are (309–330) illustrated in yellow square and Ab-359 residues are (359–382) represented in the orange square. The pink gradient colors represent the conserved unique residues 511–524, 533–544 in erythrovirus (Fig. 2).

### Phylogenetic analyses

**Fig. 3:**
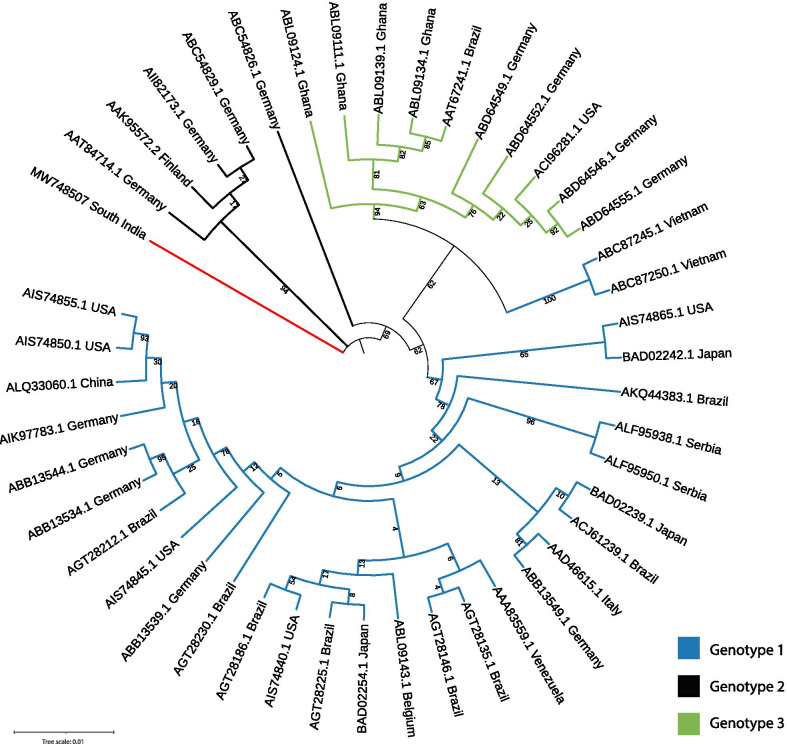
Phylogenetic tree of VP1/2 gene of human parvovirus B19V from different geographical locations, constructed using maximum likelihood method. The tree branches were colored according to the genotypes, and the South-Indian B19V strain branch is represented in red

The evolutionary selection pressure analysis indicates that the human B19V is under positive selection (ω > 0), where codon site (323) is showing evidence for positive selection (Additional file 4: Table S3). Maximum likelihood phylogeny of VP1/2 full-length sequences from global strains and South-Indian strains resolved into well-established clades. Genotypes 1, 2, and 3 are formed monophyletic clades in the phylogeny except for two strains. The South Indian strain formed a distinct clade closer to Genotype 2 strains (Fig. 3), indicating that the south-Indian strain might have diverged from Genotype 2 strains by accumulating the mutations. Interestingly, the geographically distinct strains are not monophyletic in the phylogeny, indicating different genotypes were circulating globally.

## Discussion

Here we report a distinct genotype of the human B19 virus from South India through our screening, detection, sequencing, and phylogenetic analysis of the B19V genome from suspected clinical samples.

In our study, parvovirus B19 infection was shown among 33 of 456 patients when tested by nPCR; 30 among these were also positive by qPCR and were subsequently confirmed by sequencing 75% nPCR from patients with age. ≥ 50 years respectively (Additional file 1: Table S1). In terms of age, the prevalence of Parvovirus B19 infection is 15%, 50% and 85% in pre-school children, adults and elderlies, respectively [32, 33].

Parvovirus B19 infection has spread across the globe and has been reported from all geographical regions. Different studies have emphasized the high prevalence of this disease than commonly estimated, and its seroprevalence can vary depending on age and geographical location [34]. Another point to consider is that the earlier study was probably conducted in a period that did not include an epidemic year for B19V [35, 36]. Therefore, the application of PCR has a higher diagnostic value and can help reach definite results. The present study's superiority is simultaneous conductance of PCR tests which can dramatically enhance the diagnostic power.

B19V genotypes and subtypes have a high rate of synonymous to non-synonymous nucleotide changes per site, suggesting that NS1 and VP1/2 regions are under strong purifying selection [37]. Other specific mutations (V21T, Q220E, T227S, N323S, M389I, S32T) were seen in the South-Indian strain, which is also present in Genotype 1,2 and 3 strains. These above-mentioned mutations might significantly influence other vital residues of the protein; for example, the previously reported GH loop (residues 298–328) is essential for the immunodominance properties of parvovirus [38]. In the residues of an above-stated loop that governs immunodominance in human B19V, residual mutation N323S was observed. This substitution of Asparagine (Asp) to Serine (Ser) at position 323 may be resulting in sustained angiogenesis and cell proliferation [38].

Scientists have found a very high rate of nucleotide substitutions comparable to the substitution rates of ssRNA viruses and, on average, 1–2 × 10^−4^ substitutions/site/year [39]. This significantly high mutation rate might contribute to an efficient adaptive nature of the virus, and thus, for their wide variety of cellular tropism in humans. Therefore, this virus is thought to be evolved rapidly. But a recent publication has shown the existence of B19 in the human population present around 7000 years ago [40]. Therefore, the study shows that the virus had a much lower mutation rate in its long-ago ancestors. Though the reason behind the acceleration in the mutation rate is not known, it is inferred that the virus is going through a rapid change in recent times. The small peptide of 60–100 amino acids from the VP-1 protein sequence has the maximum antibody-producing ability and can be used as a peptide vaccine [41]. These higher substitution rates might affect the virus' tropism for different host cells and account for its persistence; thereby affecting the vaccination strategies against it.

The novel mutations we observed in our study may significantly affect selection pressure and exemplifies the crucial role of VP1 protein in the viral life cycle, especially the enzymatic function of B19V phospholipase A2 (vPLA2) with diversifying substitutions, critical for long-term persistent infection. One of the novel mutations, I283V in the South Indian B19 isolate, was seen closer to its known antigenic determinant sites. This might help the virus evade host neutralizing antibodies**.** However, in our study, there were positive and negative selected sites; this could be either the inherent nature of the strains or the result of a smaller number of samples sequenced. A further diverse set of samples needs to be analyzed, and in-depth studies requires to be performed on a more extensive collection of samples.

## Conclusion

This study reports genetic and phylogenetic characteristics of human B19V strain isolated from the Southern part of India. Maximum likelihood phylogeny shows that the South-Indian strain is forming a distant clade. Besides, the South-Indian strain has three novel mutations that might significantly impact the stability and virulence of the B19V virus circulating in South-India. Further studies need to be performed to understand the functional implication of variation present in the South-Indian B19V strain. Our study helps better understand the genetic and evolutionary characteristics of South Indian isolates where positive selection pressure acting on VP1/2 could increase its viral survival and replication capabilities.

## Supplementary Information

**Additional file 1: Table S1**. List of oligonucleotide primers used for amplifying B19V NS1, VP1/2 gene. **Table S1.1.** Clinical manifestation versus molecular diagnosis.

**Additional file 2: Figure S1.** Representative amplification curves obtained to quantify viral load of patient samples using known concentrations of quantification standards (QS1-QS4) and probes specific for human parvovirus B19.

**Additional file 3: Table S2.** List of different VP1/2 isolates of parvovirus B19V from different geography used for comparative analysis.

**Additional file 4: Table S3.** Predicted immunogenicity score of THR122, ILE283 of VP1/2 protein of B19V.

## Data Availability

The data generated and analyzed in this study are available in the NCBI Genbank & SRA database under the accession number MW748507 and BioProject number PRJNA715182, respectively.

## Abbreviations

B19V: Human parvovirus B19V
HyPhy: Hypothesis Testing using Phylogenies
IU: International unit
ml: Milliliter
NCBI: National Center for Biotechnology Information
VP1/2: Viral protein 1/2
VP1u: VP1-unique region
