# Supplementary material for: Molecular-genetic characterization of human parvovirus B19 prevalent in Kerala State, India
Source: Virol J. 2021 May 5;18:96. doi: 10.1186/s12985-021-01569-1 (PMC8097873; doi:10.1186/s12985-021-01569-1)
Supplement: Supplementary file 1 — Additional file 1: Table S1. List of oligonucleotide primers used for amplifying B19V NS1, VP1/2 gene. Table S1.1. Clinical manifestation versus molecular diagnosis. [file 12985_2021_1569_MOESM1_ESM.docx]

| Primer name (gene) | Primer sequence (5’-3’) | Location | Genome position | Expected size (base pairs) |
| --- | --- | --- | --- | --- |
| B19 F | AATACACTGTGGTTTTATGGGCCG | NS1 | 1399-1422 | 284 |
| B19 R | CCATTGCTGGTTAACCACAGGT | NS1 | 1659-1682 |  |
| B19 NF | AATGAAAACTTTCCATTTAATGATGTAG | NS1 | 1495-1525 | 103 |
| B19 NR | CTAAAATGGCTTTTGCAGCTTCTAC | NS1 | 1576-1600 |  |
| PF | CAGTTATCTGACCACCCCCATGC | VP1 | 2699-2721 | 227 |
| PR | GCCAGTTGGCTATACCTAAAGTCAT | VP1 | 2925-2901 |  |
| P7 F | TGCAGAAGCCAGCACTGGTGCA | VP1/2 | 3142-3163 | 138 |
| P9 R | GTGCTCTGGGTCATATGGAAT | VP1/2 | 3260-3280 |  |
| P7 F | TGCAGAAGCCAGCACTGGTGCA | VP1/2 | 3142-3163 | 445 |
| P11 R | ATGGTCTACTAACATGCATAGGC | VP1/2 | 3567-3589 |  |
| P8 F | ATTCCATATGACCCAGAGCAC | VP1/2 | 3260-3280 | 322 |
| P11 R | ATGGTCTACTAACATGCATAGGC | VP1/2 | 3589-3567 |  |
| P10 F | GCCTATGCATGTTAGTAGACCAT | VP1/2 | 3567-3589 | 671 |
| P14 R | CACTCCTTGCTGATACTCTTTGTC | VP1/2 | 4214-4237 |  |
| P12 F | CAGCCATACCACCACTGGGACA | VP1/2 | 4127- 4148 | 111 |
| P14 R | CACTCCTTGCTGATACTCTTTGTC | VP1/2 | 4214-4237 |  |
| P12 F | CAGCCATACCACCACTGGGACA | VP1/2 | 4127- 4148 | 563 |
| P16 R | AGTACATATGGTAAATGACCTGCTG | VP1/2 | 4665-4689 |  |
| P13 F | GACAAAGAGTATCAGCAAGGAGTG | VP1/2 | 4214-4237 | 476 |
| P16 R | AGTACATATGGTAAATGACCTGCTG | VP1/2 | 4665-4689 |  |
| P12 F | CAGCCATACCACCACTGGGACA | VP1/2 | 4127- 4148 | 698 |
| P17 R | TTACGCATCCTGGCTGAGGGCA | VP1/2 | 4803- 4824 |  |
| P13 F | GACAAAGAGTATCAGCAAGGAGTG | VP1/2 | 4214-4237 | 610 |
| P17 R | TTACGCATCCTGGCTGAGGGCA | VP1/2 | 4803-4824 |  |

**Table S1:** List of Oligonucleotide primers used for amplifying the B19 NS1, VP1/2 gene

**Table S1.1:** Clinical manifestations versus molecular diagnosis

|  | **N=456** | | |
| --- | --- | --- | --- |
|  | **nPCR**  **N=33** | **qPCR**  **N=30** | **No virus**  **N=423** |
| **Age(years)** |  |  |  |
| ≤6 | 8(24.3) | 7(23.4) | 112(26.5) |
| ≥50 | 25(75.7) | 23(76.6) | 311(73.5) |
| **Clinical diagnosis** |  |  |  |
| Acute lymphoblastic leukemia | 12(36) | 11(36) | 223(52) |
| Aplastic anemia | 3(9) | 4(13) | 130(30) |
| Meningitis | 12(36) | 10(33) | 395(93) |
| pyrexia | 3(8) | 2(6) | 256(60) |
| Encephalitis | 14(42) | 12(40) | 147(34) |
| Liver failure | 7(21) | 6(20) | 230(54) |
| Multi organ failure | 8(24) | 4(13) | 268(63) |
| Hepatitis | 4(12) | 2(6) | 291(68) |

Notes: N denotes number of samples; nPCR-nested PCR; qPCR-quantitative PCR

.
