# Supplementary material for: Molecular-genetic characterization of human parvovirus B19 prevalent in Kerala State, India
Source: Virol J. 2021 May 5;18:96. doi: 10.1186/s12985-021-01569-1 (PMC8097873; doi:10.1186/s12985-021-01569-1)

**Figure Supplementary 1**:

Representative amplification curves obtained to quantify viral load of patient samples using known concentrations of quantification standards (QS1-QS4) and probes specific for human parvovirus B19


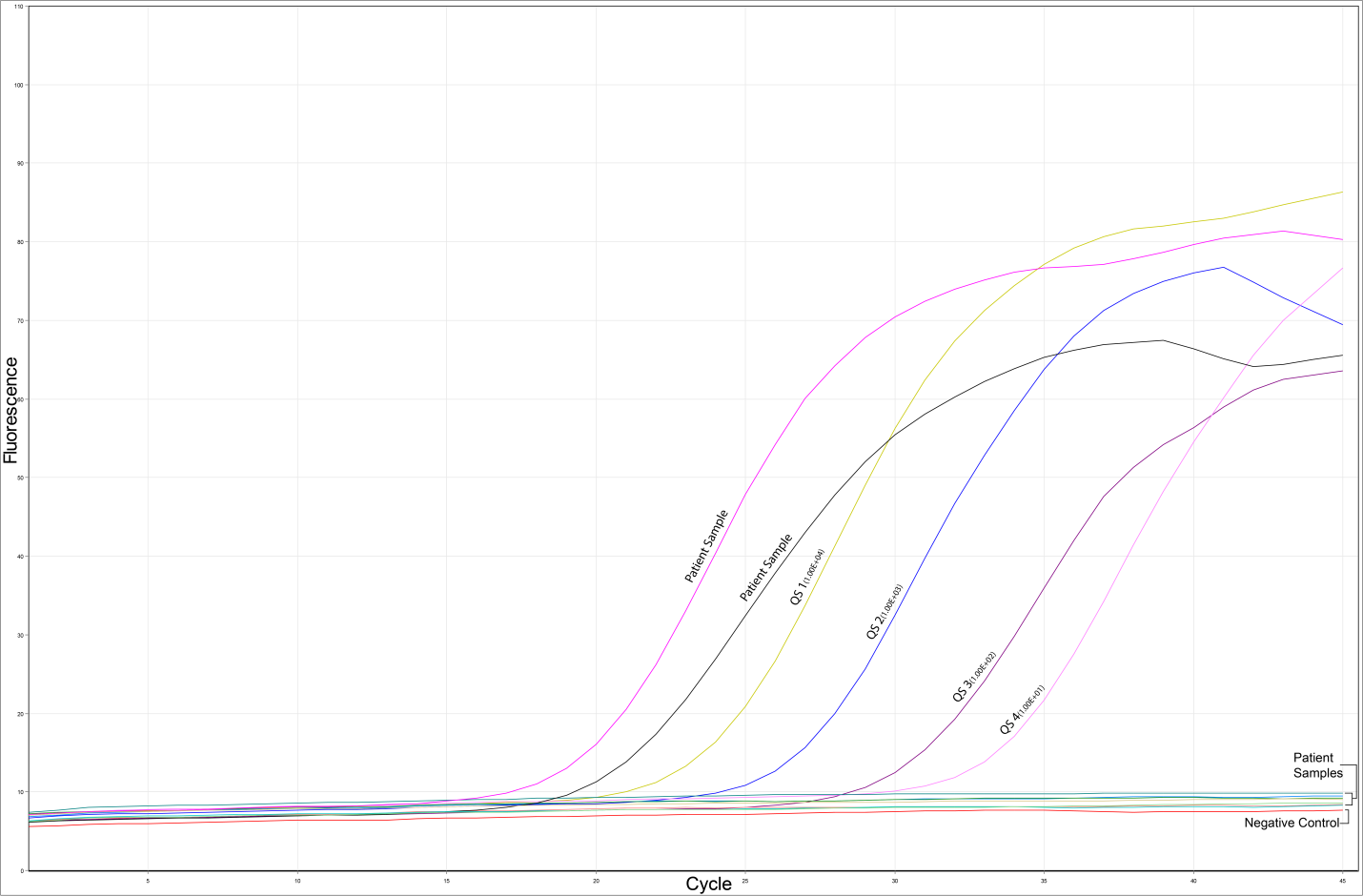

Supplement: Supplementary file 2 — Additional file 2: Figure S1. Representative amplification curves obtained to quantify viral load of patient samples using known concentrations of quantification standards (QS1-QS4) and probes specific for human parvovirus B19. [file 12985_2021_1569_MOESM2_ESM.docx]
