# Supplementary material for: Molecular-genetic characterization of human parvovirus B19 prevalent in Kerala State, India
Source: Virol J. 2021 May 5;18:96. doi: 10.1186/s12985-021-01569-1 (PMC8097873; doi:10.1186/s12985-021-01569-1)
Supplement: Supplementary file 3 — Additional file 3: Table S2. List of different VP1/2 isolates of parvovirus B19V from different geography used for comparative analysis. [file 12985_2021_1569_MOESM3_ESM.docx]

**Table S2**: List of different VP1/2 isolates from different geography used for comparative analyses

| Country | NCBI Accession id | Isolate | Genotype |
| --- | --- | --- | --- |
| Belgium | ABL09143 | C39 | 1 |
| Belgium | CBI63309 | C39_01 | 1 |
| Brazil | AAT67241 | Br543 | 3b |
| Brazil | ACJ61239 | Br05 | 3 |
| Brazil | KT268312 | B19V/RJ2929 | 1 |
| Brazil | AGT28255 | 10BRSP2230 | 1 |
| Brazil | AGT28238 | 10BRSP1090 | 1 |
| Brazil | AGT28230 | 09BRSP2002 | 1 |
| Brazil | AGT28225 | 07BRSP7611 | 1 |
| Brazil | AGT28212 | 07BRSP7044 | 1 |
| Brazil | AGT28186 | 10BRSP2014 | 1 |
| Brazil | AGT28179 | 09BRSP0883 | 1 |
| Brazil | AGT28171 | 09BRSP6081 | 1 |
| Brazil | AGT28161 | 10BRSP2440 | 1 |
| Brazil | AGT28151 | 10BRSP0109 | 1 |
| Brazil | AGT28146 | 09BRSP0491 | 1 |
| Brazil | AGT28135 | 09BRSP6896 | 1 |
| Brazil | AGT28106 | 09BRSP2330 | 1 |
| Brazil | AGT28102 | 08BRSP4111 | 1 |
| Brazil | AGT28085 | 09BRSP6547 | 1 |
| Brazil | AGT28073 | 10BRSP0150 | 1 |
| Brazil | AGT28062 | 07BRSP7612_1A | 1 |
| China | ALQ33060 | HZ1 | 1 |
| Finland | AY044266 | Lali | 2 |
| Germany | ABD64555 | BN303 | 3 |
| Germany | ABD64552 | BN60 | 3 |
| Germany | ABD64549 | BN59 | 3 |
| Germany | ABD64546 | BN58 | 3 |
| Germany | AAT84698 | D2 | 2 |
| Germany | AAT84714 | C2 | 2 |
| Germany | AAT84711 | G2 | 2 |
| Germany | AAT84708 | B2 | 2 |
| Germany | ABC54829 | BN33 | 2 |
| Germany | ABC54826 | BN32 | 2 |
| Germany | ABC54823 | BN31 | 2 |
| Germany | CAG30506 | Human serum | 2 |
| Germany | KM065414 | WHO_IRP | 1 |
| Germany | KM065415 | EQDM_BRP | 1 |
| Germany | AII82176 | BB2 | 1 |
| Germany | AII82173 | BB1 | 1 |
| Germany | ABD64543 | BN30 | 1 |
| Germany | ABB13549 | AnTo | 1 |
| Germany | ABB13544 | OsFr | 1 |
| Germany | ABB13539 | SN807 | 1 |
| Germany | ABB13534 | Kyma | 1 |
| Ghana | ABL09111 | Gh2768 | 3b |
| Ghana | ABL09139 | R0748 | 3b |
| Ghana | ABL09116 | Gh3051 | 3a |
| Ghana | ABL09134 | R0693 | 3 |
| Ghana | ABL09129 | D1599 | 3 |
| Ghana | ABL09124 | R0277 | 3 |
| Ghana | ABL09120 | R0416 | 3 |
| Italy | AF162273 | HV | 1 |
| Japan | AB126264 | AN30 | 1a |
| Japan | AB126265 | AN34 | 1a |
| Japan | AB126269 | AN66 | 1a |
| Japan | AB126263 | AN28 | 1 |
| Serbia | ALF95961 | MJ21 | 1 |
| Serbia | ALF95950 | 5248 RS6 | 1 |
| Serbia | ALF95944 | 3341 RS4 | 1 |
| Serbia | ALF95938 | 1577 RS7 | 1 |
| UK | AY504945 | Nan | 1 |
| UK | AAR97627 | NAN | 1 |
| US | AY386330 | J35 | 1 |
| USA | ACI96281 | P1 | 3 |
| USA | AIS74865 | KU12 | 1 |
| USA | AIS74860 | KU11 | 1 |
| USA | AIS74855 | KU8 | 1 |
| USA | AIS74850 | KU5 | 1 |
| USA | AIS74845 | KU4 | 1 |
| USA | AIS74840 | KU3 | 1 |
| USA | AIS74835 | KU2 | 1 |
| USA | ACL36596 | KU1 | 1 |
| USA | KX752821 | 3rd_WHO_IRP | 1 |
| Venezuela | AAA83559 | VEN1 | 1 |
| Vietnam | ABC87250 | Vn115 | 1b |
| Vietnam | ABC87245 | Vn147 | 1 |
