# Supplementary material for: Molecular-genetic characterization of human parvovirus B19 prevalent in Kerala State, India
Source: Virol J. 2021 May 5;18:96. doi: 10.1186/s12985-021-01569-1 (PMC8097873; doi:10.1186/s12985-021-01569-1)
Supplement: Supplementary file 4 — Additional file 4: Table S3. Predicted immunogenicity score of THR122, ILE283 of VP1/2 protein of B19V. [file 12985_2021_1569_MOESM4_ESM.docx]

**Table S3**: List of residual mutations in VP1/2 sequence of parvovirus B19V changing overall stability of the VP1/2 structure

| Mutation | Predicted ∆∆G (kcal/mol) | RSA | Destabilizing/Stabilizing |
| --- | --- | --- | --- |
| Val(21)Thr | -0.563 | 61 | Destabilizing |
| Ser(32)Thr | -0.396 | 45 | Destabilizing |
| Thr(122)Ala | -0.73 | 25 | Destabilizing |
| Val(128)Ile | -1.212 | 0 | Destabilizing |
| Gln(220)Glu | 0.068 | 41 | stabilizing |
| Thr(227)Ser | -0.438 | 29 | Destabilizing |
| Asp(323)Ser | -0.04 | 81 | Destabilizing |
| Ile(283)Val | -0.629 | 30 | Destabilizing |
| Met(389)Ile | -0.194 | 53 | Destabilizing |

Notes: ∆∆G, Gibbs-free energy change upon mutation, RSA: Relative solvent accessible area.
